# High magnetoelectric coupling of Metglas and P(VDF-TrFE) laminates

**DOI:** 10.1038/s41598-022-09171-3

**Published:** 2022-03-28

**Authors:** Henrik Staaf, Anurak Sawatdee, Cristina Rusu, David Nilsson, Philipp Schäffner, Christer Johansson

**Affiliations:** 1 RISE Research Institutes of Sweden, Sensors and Materials, Göteborg, Sweden; 2 RISE Research Institutes of Sweden, Printed Electronics, Norrköping, Sweden; 3 Joanneum Research Forschungsgesellschaft mbH, MATERIALS-Institute for Surface Technologies and Photonics, Weiz, Austria

**Keywords:** Techniques and instrumentation, Electrical and electronic engineering, Sensors and biosensors

## Abstract

Magnetoelectric (magnetic/piezoelectric) heterostructures bring new functionalities to develop novel transducer devices such as (wireless) sensors or energy harvesters and thus have been attracting research interest in the last years. We have studied the magnetoelectric coupling between Metglas films (2826 MB) and poly(vinylidene fluoride-trifluoroethylene) (P(VDF-TrFE)) in a laminate structure. The metallic Metglas film itself served as bottom electrode and as top electrode we used an electrically conductive polymer, poly(3,4-ethylene-dioxythiophene):poly(styrene sulfonate) (PEDOT:PSS). Besides a direct electrical wiring via a graphite ink, a novel contactless readout method is presented using a capacitive coupling between the PEDOT:PSS layer and an electrode not in contact with the PEDOT:PSS layer. From the experimental result we determined a magnetoelectric coupling of 1445 V/(cm·Oe) at the magnetoelastic resonance of the structure, which is among the highest reported values for laminate structures of a magnetostrictive and a piezoelectric polymer layer. With the noncontact readout method, a magnetoelectric coupling of about 950 V/(cm·Oe) could be achieved, which surpasses previously reported values for the case of direct sample contacting. 2D laser Doppler vibrometer measurements in combination with FE simulations were applied to reveal the complex vibration pattern resulting in the strong resonant response.

## Introduction

The magnetoelectric (ME) coefficient of a composite/laminate structure is the ratio between the response electric field and the applied magnetic excitation field^1^. For a laminate structure, the magnetoelectric coupling mechanism is mediated via the strain induced in the magnetostrictive material by an external magnetic field, which translates into the piezoelectric layer at the laminate interface and is converted to an electric field due to the inverse piezoelectric effect^2^. With electrodes applied to the piezoelectric layer, the electric field can be measured as a voltage between the electrodes. The magnetoelectric coupling effect can be used in different applications such as for energy harvesting^3–6^ and as sensitive magnetic field sensors^2,7^. In the ME devices different design approaches (layers or nanocomposites of the piezoelectric and magnetostrictive materials) and material combinations are being used^8,9^.

Among the magnetostrictive materials that can be used, Metglas is very high performing. It is an amorphous iron-nickel based material with good dynamic magnetic properties and high magnetoelastic coupling^2^ and has been successfully combined with piezoelectric polymers such as Polyvinylidene fluoride (PVDF), its copolymer P(VDF-TrFE) or Poly(vinylidene fluoride-hexafluoropropylene) (P(VDF-HFP)), as laminated structures^2,10–12^. As previously mentioned, it is also possible to use nanostructured magnetic composites as the magnetostrictive material by using for instance nanoparticles of CoFe_2_O_4_ integrated in P(VDF-TrFE) films^13^, in PVDF microspheres^14^ or in PVDF and P(VDF-TrFE) matrixes^15^. Also, nanoparticles of Fe_3_O_4_ integrated in P(VDF-TrFE) has been used in ME coupling devices^16^. Other magnetoelectric coupling studies on composite structures has been performed earlier on different material combinations by using ceramic materials like lead zirconate titanate (PZT) as the piezoelectric material and Ni/TbFe2^17^ and Terfenol-D^18^ as the magnetostrictive material. All of these previous magnetoelectric studies show a maximum magnetoelectric coupling coefficient of about 7 V/(cm·Oe) in the non-resonant mode and about 370 V/(cm·Oe) in a resonant mode.

In this study, we use a Metglas 2826 MB film as the magnetostrictive layer in combination with screen-printed P(VDF-TrFE) on top as the piezoelectric layer. We measured the magnetic coupling coefficient both in non-resonant and resonant mode with physical contact to the laminate ME sample using a PEDOT:PSS layer in contact with the P(VDF-TrFE) layer, as well in a non-contact capacitive mode that has previously been used in other piezoelectric sensor applications^19^. In a magnetostrictive film it is possible to obtain a magnetoelastic resonance at a specific resonance frequency, where an elastic standing wave is built up in the film^2,20–22^. The resonance frequency is dependent on elastic modulus, shape and dimension of the film as wells as on the clamping conditions. We have used this magnetoelastic resonance to measure the ME laminate structure in resonant mode as well as in the non-resonant mode. The Metglas film of length 20 mm was clamped in the middle of the film. 2D scanning laser Doppler vibrometer measurements were applied to obtain the full vibration pattern. Measurements were also compared with FEM simulations. To the best of our knowledge, the obtained magnetoelectric coupling coefficients at magnetoelastic resonance are the highest reported so far.

## Experimental and methods

**Figure 1:**
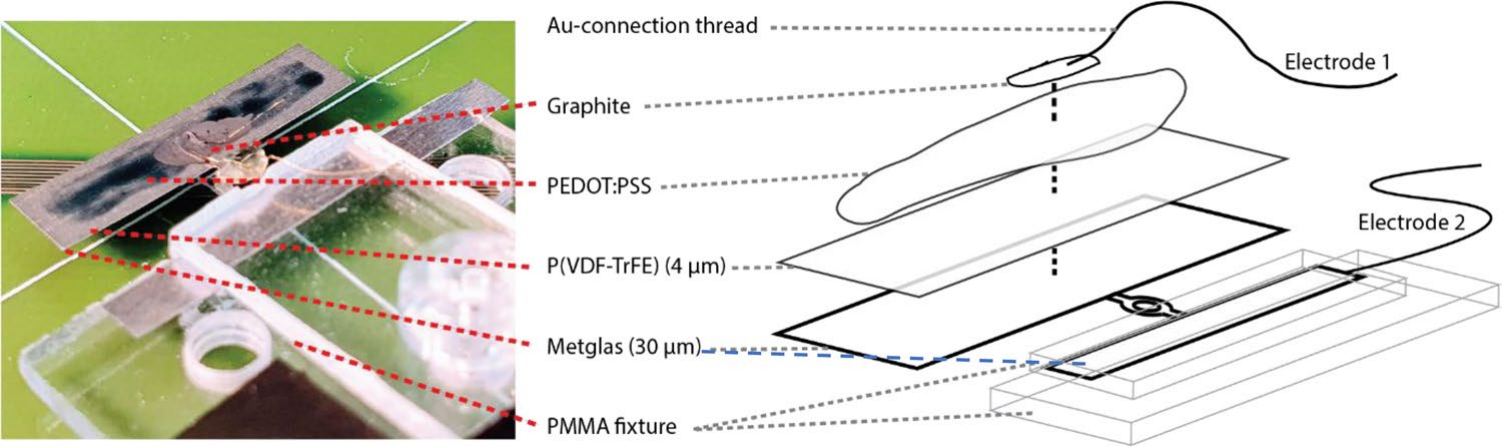
Sample setup showing the laminate structure comprised of Metglas film (magnetostrictive), P(VDF-TrFE) layer (piezoelectric) and PEDOT:PSS layer (conductive polymer) with a graphite dot to attach the thin Au wire (electrode 1). The second electrode (electrode 2) is connected to the metallic Metglas film that is clamped by the PMMA structure. For the non-contact measurements (capacitive coupling) an electrode was placed at various distances above the PEDOT:PSS layer, and electrode 2 was connected with contact to the clamped Metglas film to the electronics (see Fig. 3).

The Metglas 2826 MB films (with chemical structure Fe_40_Ni_38_Mo_4_B_18_) were purchased from Metglas Incorporated with a ca. 30 µm thickness. The films were further etch-processed to a structure consisting of two areas/parts: the measuring part of 20 mm length and 5 mm width and the clamping part, which are connected via a narrow bridge section (Fig. 1). The piezoelectric layers were manufactured by depositing a layer of P(VDF-TrFE) on the measuring part of the Metglas structure by means of screen printing. Piezotech FC 20 (purchased from Piezotech Arkema) was used as the piezoelectric ink in the screen printing process, which has a VDF/TrFE molar ratio of 80/20 mol%. The whole area of the Metglas film was deposited with P(VDF-TrFE). On top of the P(VDF-TrFE) layer PEDOT:PSS (Clevios™ S V3) was deposited, leaving about 1 mm margins with respect to the P(VDF-TrFE) edges. The PEDOT:PSS layer made up the top electrode, whereas the conductive Metglas itself formed the bottom electrode. A thin Au wire was connected to the PEDOT:PSS layer with a graphite ink. After the P(VDF-TrFE) and PEDOT:PSS/graphite deposition, the piezoelectric layer was electrically poled by applying a high voltage between the two electrodes and the applied poling voltage was swept to obtain the polarization curve. The voltage was a triangle way with a periodic time of 400 ms and a maximum voltage of 450 V. The poling was carried out at 295 K. From the polarization curve determined from the poling process a remanence polarization of about 7.4 µC/cm^2^ and a coercivity electric field of 48 MV/m could be determined, which are typical literature values of PVDF-TrFE^23^. The piezoelectric coefficient of the piezoelectric laminate was determined by applying a calibrated force in the thickness and length direction. The piezoelectric charge coefficients were determined to be *d*_*33*_ = − 23 pC/N (and a piezoelectric voltage coefficient of *g*_*33*_ = − 0.22 Vm/N) and *d*_*31*_ = 7 pC/N (and a piezoelectric voltage coefficient of *g*_*31*_ = 0.07 Vm/N), values that are in good agreement with data from the supplier of the piezoelectric ink (Piezotech Arkema). The capacitance of the piezoelectric laminate layer was measured using a Hameg HM8118 LCR meter. The thickness of the P(VDF-TrFE) laminate was measured by a calliper with micrometre resolution and the reading agreed well with the value obtained from the polarization curve when comparing the coercive field strength of P(VDF-TrFE) with the corresponding voltage in the poling process. The P(VDF-TrFE) thickness was determined to be 4 µm. The thickness of the piezoelectric layer was used to determine the coercivity electric field from the polarization curves and the value is in good agreement with earlier studies on P(VDF-TrFE)^23^ that confirms the determined P(VDF-TrFE) thickness. The capacitance of the piezoelectric laminate structure was also measured, and the determined permittivity (using a P(VDF-TrFE) thickness of 4 µm) was equal to 10 that agrees well with the data from the supplier (Piezotech Arkema) of the piezoelectric ink. This further confirms the determined P(VDF-TrFE) thickness. The Metglas/P(VDF-TrFE)/PEDOT:PSS laminate structure was fixed to the sample holder (a PMMA structure) with the clamping part and aligned laterally so that the symmetry axis of the Metglas film, corresponding to the node of the first magnetoelastic resonance mode, was in the centre. A photo with associated schematic picture of the sample setup is presented in Fig. 1.

In order to magnetically excite and measure the dynamic magnetic response from the laminate structure, we used a magnetic AC susceptibility (ACS) measurement system built in our lab specially designed for magnetic films. It is based on two 2D in-plane coils for magnetic field excitation (the AC magnetic field) and detection, where the detection coil is rotated by 90° with respect to the excitation coil to cancel excitation field and thus only measures the response from the magnetic film. We placed a magnet sheet beneath the excitation and detection coils that field biased the Metglas film (applying the DC magnetic field). The bias magnet was tuned to give an optimum magnetic response from the Metglas film with the length (20 mm) that we are using in this study. The static bias field at the Metglas film was 0.68 mT. The DC magnetic field pointed in the same direction as the AC magnetic field. We also used dedicated electronics that excited the excitation coils and collected the induced voltage, which is dependent on the amplitude and phase of the AC susceptibility of the sample, from the detection coil. The whole measurement setup is software controlled. The DC and AC fields at the sample position were measured with calibrated commercial magnetic sensors (Lakeshore F71 Teslameter).

During the measurements we measure and control the current that enters the excitation coil. For the used current settings in the experiment, we have an AC magnetic field amplitude of 0.0035 mT (0.035 Oe) at the sample position.

The clamping part of the Metglas film and the thin Au-wire form the two electrodes to the P(VDF-TrFE) layer (Fig. 1, schematic). Both electrodes were connected differentially to a high impedance amplifier (Stanford Research Systems, model SR560, input impedance 100 MΩ) and from the amplifier output to the data collection device (Agilent Infinium 1 Gs/s oscilloscope), using the excitation current as a reference signal. From the measured piezoelectric voltage we determined both the amplitude and the phase relative to the applied magnetic field. The magnetoelectric coupling, *α*_*ME*_, is calculated according to^2,17^

1 $$ \alpha_{ME} = \frac{dE}{{dH}} = \frac{{V_{piezo} }}{{tH_{ac} }} $$

where *E* is the electric field in the P(VDF-TrFE) layer, *V*_*piezo*_ is the measured piezoelectric voltage, *t* is the thickness of the P(VDF-TrFE) layer and *H, H*_*ac*_ is the magnetic AC field and magnetic field amplitude, respectively, applied to the sample. The measured capacitance of the P(VDF-TrFE) layer, *C* = 970 pF, together with the amplifier input impedance, *R* = 100 MΩ, gives a piezoelectric cut-off frequency at 100 kHz of $1/(2\pi RC)$ ≈ 1.5 Hz. This is well below the excitation frequencies, meaning that we measure the maximum piezoelectric signal, *V*_*piezo*_, without any phase changes of the piezoelectric signal.

We also used laser Doppler vibrometry (LDV) measurements (Polytec OFV-056) during excitation of the Metglas/P(VDF-TrFE) laminate structure in order to study any bending modes of the film. Since the laser beam was oriented perpendicular to the surface of the Metglas/P(VDF-TrFE) film, we only measured the vibrations normal to the film surface.

## Results and discussion

**Figure 2:**
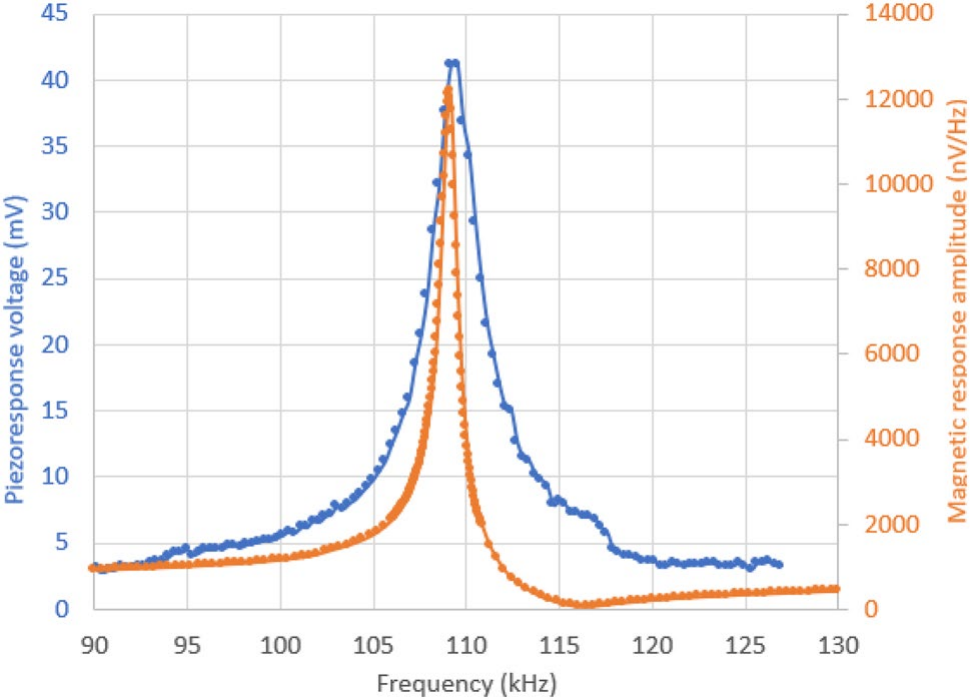
Piezoelectric peak-to-peak voltage (blue) and magnetic amplitude signal response (orange) versus frequency of the magnetic field excitation. Since we measure the magnetic response from the induction voltage from the detection coil, we normalize the magnetic signal with frequency in order to obtain the AC susceptibility magnitude. The contacts to the amplifier are physically connected to the sample (Metglas clamping and Au-wire).

Several samples were measured and in Fig. 2 the magnetic signal and piezoelectric voltage are shown versus the excitation frequency when connecting the electrodes directly to one of the Metglas/P(VDF-TrFE) laminate samples. Good reproducibility was obtained when comparing the results from the samples. When presenting the analysis result, we also give the uncertainties in the measured values and analysis results as obtained from measurements from the other samples and the uncertainties of the measured values.

From Fig. 2 we can see the effect of the magnetoelastic resonance in the frequency range of 105 kHz and 120 kHz. The magnetoelastic resonance effect in magnetostrictive films and its applications have been studied and reported earlier^2,20–22^. There is a large increase in the magnetic response at about 109 kHz where the magnetoelastic resonance is located, and it drops down to almost zero at 115 kHz where the antiresonance is located and then the magnetic response signal increase again with increasing frequencies. At the magnetoelastic peak resonance frequency of 109 kHz we have the first mode of an elastic standing wave with a node in the middle of the film. This mode gives maximum lateral displacements at the ends of the film as well as the largest magnetic response. At the resonance peak frequency, lateral strain amplitudes in the laminate structure are at maximum, which translates into the P(VDF-TrFE) layer and results in a maximum piezoelectric voltage from the piezoelectric layer, as we can see in Fig. 2. From the maximum piezoelectric amplitude voltage (20.2 ± 0.5 mV), where the voltage amplitude is determined by dividing the peak-to-peak voltage in Fig. 2 by 2, we can determine the magnetoelectric coupling coefficient at resonance by Eq. (1) using the thickness of the piezoelectric laminate (4 ± 0.2 µm) and the AC magnetic field amplitude (0.035 ± 0.001 Oe), taking into account the uncertainties in the measured values. The non-resonant magnetoelectric coupling can also be determined by using the piezoelectric voltage outside the resonance range in the range of 90 kHz, since the Metglas material have magnetostrictive properties even when the film is not in resonance. We also measured on a P(VDF-TrFE) layer that was unpoled (non-piezoelectric active) and obtained piezoelectric voltages in the range of 1.5 mV peak-to-peak voltage which is the background signal due to induced voltages in electrodes and cables close to the detection zone. The background signal was subtracted from the measured piezoelectric response. From this result we obtain a measured magnetoelectric coupling at resonance of the structure of about 1445 ± 150 V/(cm·Oe) and about 100 ± 10 V/(cm·Oe) at non-resonance (90 kHz). The obtained coupling coefficient value at resonance is more than 4 times higher than previously reported values for Metglas type magnetostrictive (e.g. Vitrovac) and PVDF layers in laminate structures yielding ME coupling coefficients of 45–120 V/(cm·Oe)^2^ and 300 V/(cm·Oe)^10^. Further, from Fig. 2 the piezoelectric voltage peak (the elastic part) is somewhat broader than the magnetoelastic resonance response peak. This is probably due to mechanical losses in the P(VDF-TrFE) and PEDOT:PSS layer. The measured phase for the magnetic response (relative to the applied magnetic field) is about 165°, which is in good agreement with the theoretical phase shift of 170° that returns to zero phase above the resonance. The phase of the piezoelectric response (the elastic part) changes also its phase at the resonance by about 180°, according to theory^24,25^. This shows that the piezoelectric voltage below and above the resonance is not only a background signal but instead also originates from the magnetoelectric coupling.

**Figure 3:**
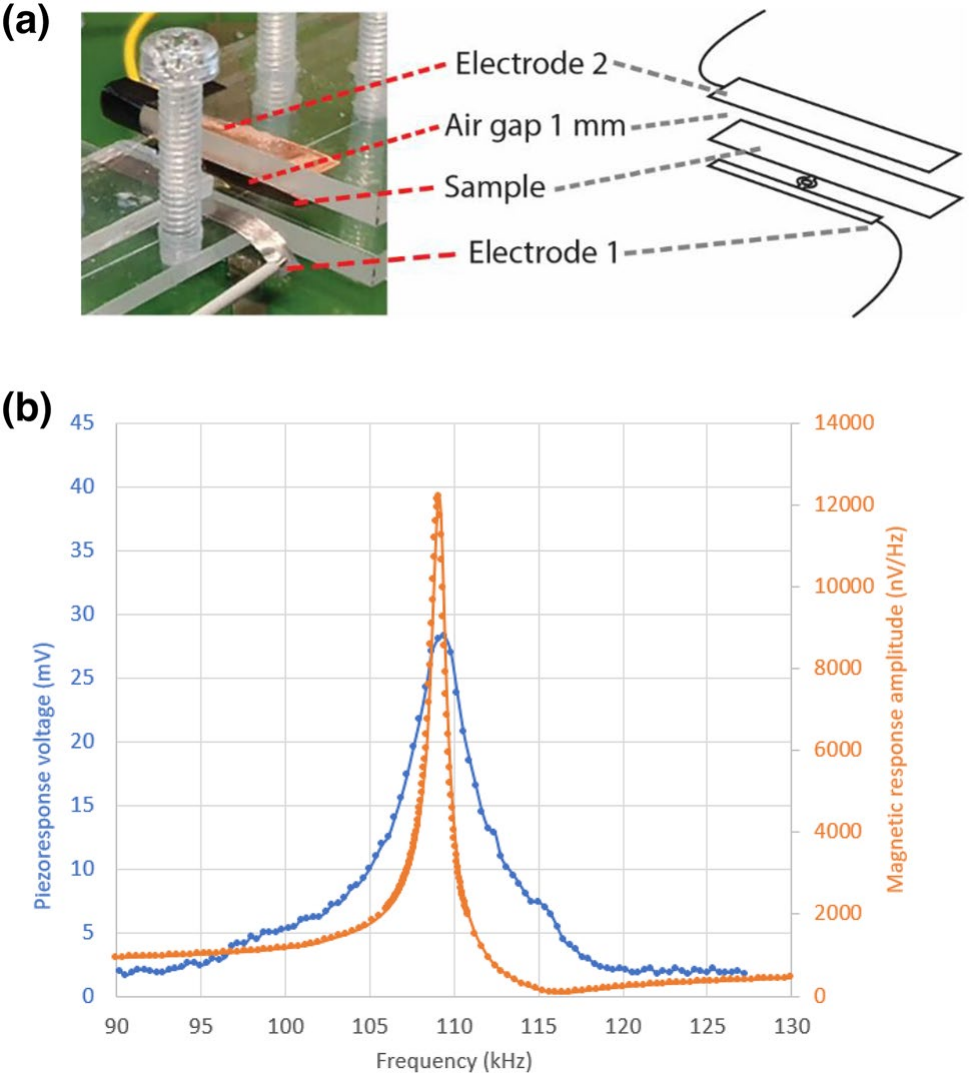
Non-contact measurement of the ME coupling. (**a**) Sample with schematic showing the airgap between the sample and electrode 2. (**b**) Piezoelectric peak-to-peak voltage (blue) and magnetic amplitude signal response (orange) versus frequency of the magnetic field excitation. The cables to the amplifier are connected to the clamped part of the Metglas film [electrode 1 in (**a**)] and to the non-contact electrode (with an area equal to the PEDOT:PSS area), at a distance 1 mm above the PEDOT:PSS layer (utilizing the capacitive coupling between PEDOT:PSS and electrode).

The result and measurement setup when using the non-contact capacitive coupling electrode above the PEDOT:PSS layer can be seen in Fig. 3.

In the experiment with non-contact measurements, we were careful that the Au-wire (that was connected to the graphite dot) did not touch the reading electrode above the PEDOT:PSS layer and not build up a close approach to the reading electrode. As can be seen in Fig. 3b even when we have a non-contact reading electrode above the PEDOT:PSS (not in physical contact with the sample), we have a piezoelectric peak-to-peak voltage of about 28 mV, giving a voltage amplitude of 14 ± 0.5 mV. The peak of the piezoelectric voltage is somewhat broader in frequency than compared with the case of direct contacting the PEDOT layer (see Fig. 2), indicating a higher damping of the system. For the non-contact readout case we obtain a magnetoelectric coupling of about 950 ± 95 V/(cm·Oe) at resonance using Eq. (1) and the thickness of the piezoelectric laminate and the field amplitude in the same way as for the contact case. This value is also higher than previously reported values using contact measurements of the sample.

As previously shown in the literature, the magnetoelectric coupling coefficient can be compensated for the demagnetization fields in the Metglas film when excited with a magnetic field^2^. In this case the internal AC magnetic field amplitude is calculated by considering the demagnetization field in the Metglas film and the determined magnetoelectric coupling coefficient will be an intrinsic value. When using data of measured magnetic properties of the used Metglas film^25^ a effective magnetic susceptibility of 1135 is obtained and a demagnetization factor for thin magnetic films^26^ together with the Metglas dimensions yielding a demagnetization factor of 0.000675, we get an internal field amplitude of 0.035·(1–0.000675·1135) = 0.008 Oe, which gives an intrinsic magnetoelectric coupling coefficient of 6200 V/(cm·Oe) using Eq. (1), at resonance for the measurements with physical contacts to the laminate sample.

**Figure 4:**
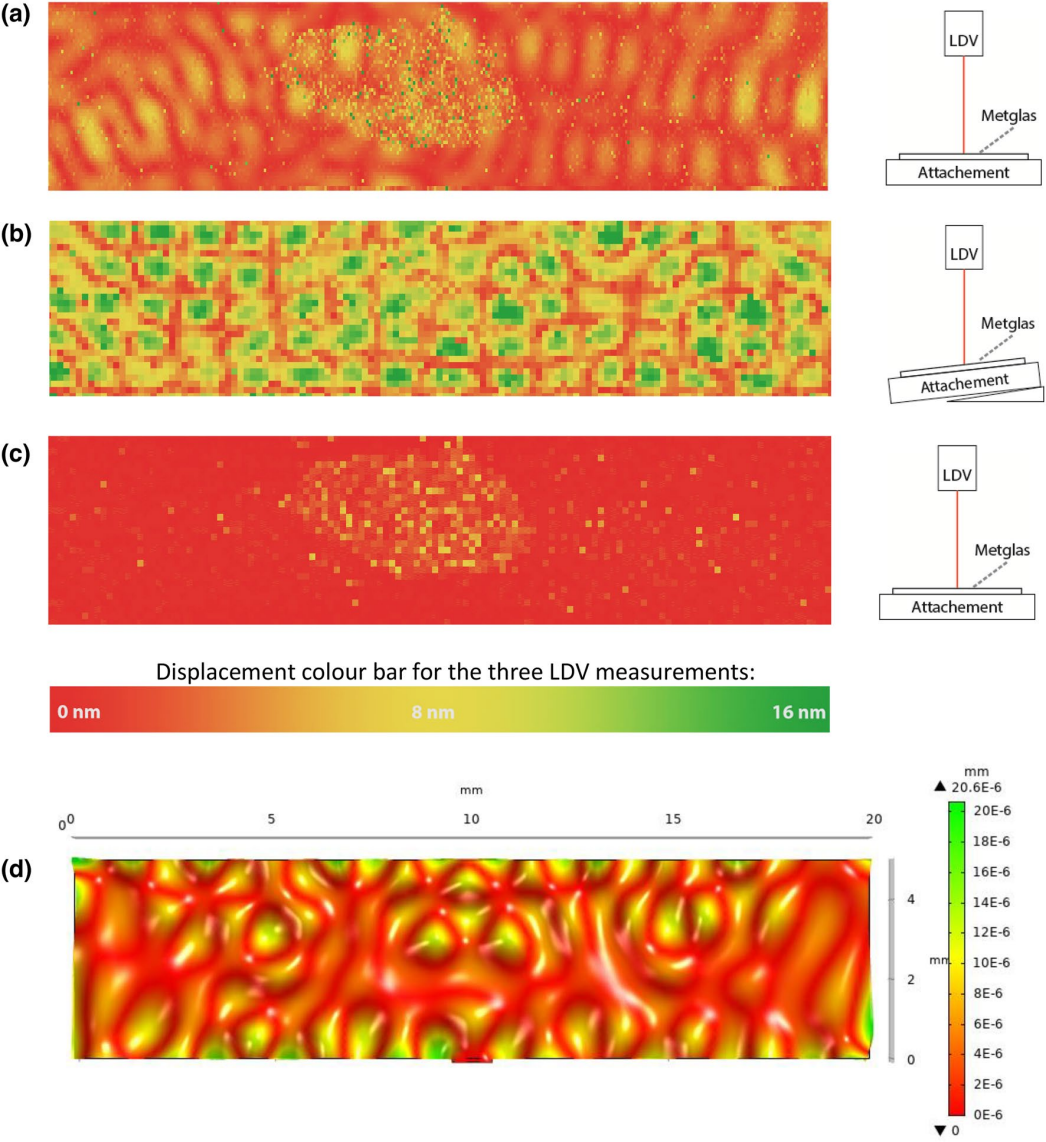
LDV measurements on vertical displacement on the laminate sample over the whole surface area top view followed by schematic side view. (**a**) The vertical displacement at the P(VDF-TrFE)/PEDOT:PSS side when the laminate sample surface is excited perpendicular at the magnetoelastic resonance frequency (109 kHz). (**b**) The vertical displacement at the P(VDF-TrFE)/PEDOT:PSS side when the laminate sample is excited at a tilted angle of 2.3° at the resonance frequency (109 kHz), to be able to get a clearer view of the surface longitude movement. (**c**) The background signal at the P(VDF-TrFE)/PEDOT:PSS side obtained with no excitation. (**d**) Simulated vertical displacement amplitudes for Metglas with a layer of PVDF to verify the magnetic mode coupling to the mechanical mode and the pattern visible from LDV.

Through LDV (laser Doppler vibrometry) we examined the vibrations in the laminated sample when it is excited by an AC magnetic field at the magnetoelastic resonance frequency of 109 kHz (Fig. 4). We also swept the frequency over the resonance frequency and measured the LDV response. We used exactly the same set up as in the previous magnetic/piezoelectric measurements for applying AC field and DC bias. The measurements were performed with the PEDOT:PSS side facing the LDV. During excitation, the LDV measured 18,090 points spread over a rectangular matrix on the laminate sample presented in Fig. 4a and measured 3810 points presented in Fig. 4b,c.

In Fig. 4a there are multiple small vertical displacement amplitudes between 0 and 8 nm (red is 0 and yellow is 8 nm) in a 2D lattice structure resulting from a complex dynamic mechanical mode in the film when the film is excited at the magnetoelastic resonance (109 kHz). This vibrational pattern might contribute to the piezoelectric signal. The horizontal lateral strains originating from the magnetoelastic resonance cannot be visualized in this LDV analysis since the setup is only sensitive to vertical displacements. The vertical displacement pattern shows that the laminate sample does not bend during excitation, as the vertical displacement does not gradually increase from the middle of the film towards the outer free ends of the film.

The first magnetic resonance mode in the film when clamping the film in the middle is a lateral node in the middle with largest lateral displacements at the ends of the film. Instead, from Fig. 4a, we can see a small vertical displacement pattern in the laminate sample with maximum vertical displacement amplitudes of about 8 nm. This pattern can be due to higher order mechanical surface vibrational modes^27,28^. Since we get a more or less zero vertical displacement without excitation, see Fig. 4c, it is not a surface roughness.

To get a better view of the longitudinal motion on the laminated sample, a wedge was placed below one side, giving the laminated sample a tilt angle of 2.3°. In Fig. 4b the vertical displacement pattern becomes clearer and the mode shapes on the surface from higher order surface modes is highly visible as green islands. Since the LDV analysis gives the vertical displacement, we can estimate the horizontal displacement since we know the tilt angle and the vertical displacement (maximum displacement of 16 nm as obtained from Fig. 4b and also taking into account the 8 nm displacement in Fig. 4a with no tilt angle). This will give a horizontal displacement of 200 nm at the magnetoelastic resonance. From the piezoelectric peak voltage amplitude at the magnetoelastic resonance (i.e. about 20 mV, see Fig. 2), the piezoelectric voltage coefficient in the length mode, the thickness of the PVDF-TrFE layer and the elasticity module of PVDF-TrFe (typical value E = 2.8 GPa), it is possible to estimate an elastic strain amplitude in the P(VDF-TrFE) and by using the dimensions of the laminate structure we get a displacement amplitude at the ends of the laminate structure in the range of 215 nm at the magnetoelastic resonance that is in good agreement with the result from the LDV analysis.

FE simulations (using COMSOL Multiphysics^®^) at an excitation frequency equal to the measured magnetoelastic resonance frequency, as presented in Fig. 4d, verify that the magnetic longitudinal mode couples to a higher order of mechanical resonance frequency.

When we swept the frequency of the magnetic AC field we could see a vertical displacement amplitude (from frequency analysis of the LDV signal) and large magnetic response when we entered the resonance frequency range (between 100 and 120 kHz), correlating to the frequency of the magnetic excitation field where we have the piezoelectric response as seen in Figs. 2 and 3. Thus, no vertical displacements were measured when exciting the Metglas/P(VDF-TrFE) composite at frequencies below or above this resonance frequency range. It is also interesting that we can see the graphite dot even in the background signal from the LDV image, see Fig. 4c.

## Conclusion

By magnetic and piezoelectric measurements we have obtained very high values of measured magnetoelectric coupling, 1445 V/(cm·Oe) at resonance, between Metglas and P(VDF-TrFE) laminates when we use PEDOT:PSS (and graphite) as the top electrode. The determined magnetoelectric coupling is higher than expected and is more than 4 times higher than previously reported for this type of magnetolectric system. We believe that the PEDOT:PSS layer as top electrode is more elastically compatible with the P(VDF-TrFE) laminate and enhances the strain in the P(VDF-TrFE) laminate as induced from the Metglas film, and thereby increases the magnetoelectric coupling. We have also shown that the measurements can be performed with a non-contact reading electrode by utilizing the capacitive coupling between the PEDOT:PSS layer and the reading electrode. By using laser Doppler vibrometry (LDV) measurements we have also observed a complex 2D vibration pattern of vertical displacements (at the resonance) related to higher order modes, which was confirmed both in literature as well by performed FE simulations using the actual film dimensions and properties of the laminate structure. The observed vibrational pattern might contribute to the high value of the magnetoelectric coupling considering the elastic PEDOT:PSS layer as the top electrode that probably also enhance the vibrational pattern.
